# Effects of temperature and photosynthetically active radiation on virioplankton decay in the western Pacific Ocean

**DOI:** 10.1038/s41598-018-19678-3

**Published:** 2018-01-24

**Authors:** Wei Wei, Rui Zhang, Lulu Peng, Yantao Liang, Nianzhi Jiao

**Affiliations:** 1 State Key Laboratory of Marine Environmental Science, College of Ocean and Earth Sciences, Institute of Marine Microbes and Ecospheres, Xiamen University, Xiamen, 361102 PR China; 2 Research Center for Marine Biology and Carbon Sequestration, Shandong Provincial Key Laboratory of Energy Genetics, Qingdao Institute of BioEnergy and BioProcess Technology, Chinese Academy of Sciences, Qingdao, 266101 PR China

## Abstract

In this study, we investigated virioplankton decay rates and their responses to changes in temperature and photosynthetically active radiation (PAR) in the western Pacific Ocean. The mean decay rates for total, high-fluorescence, and low-fluorescence viruses were 1.64 ± 0.21, 2.46 ± 0.43, and 1.57 ± 0.26% h^**−**1^, respectively. Higher temperatures and PAR increased viral decay rates, and the increases in the decay rates of low-fluorescence viruses were greater than those of high-fluorescence viruses. Our results revealed that low-fluorescence viruses are more sensitive to warming and increasing PAR than are high-fluorescence viruses, which may be related to differences in their biological characteristics, such as the density of packaged nucleic acid materials. Our study provided experimental evidence for the responses of natural viral communities to changes in global environmental factors (e.g., temperature and solar radiation).

## Introduction

The fifth report of the Intergovernmental Panel on Climate Change (IPCC) predicts that under the conditions of Representative Concentration Pathways 8.5 (RCP 8.5), global average temperatures will increase by 2.6–4.8 °C (average 3.7 °C) by the end of this century, relative to temperatures from 1986–2005^1^. This estimated global temperature change will directly warm the marine surface water and increase vertical stratification in the oceans^2^. Increased vertical stratification will then slow the mixing of surface and deep water, increasing the exposure of microorganisms in the marine surface waters to high temperatures and solar irradiation^2^. Numerous studies have investigated how changes in temperature and light irradiation will affect the physiology and ecology of marine macro-organisms and, recently, microorganisms^1^. Observatory, experimental, and modelling studies have indicated that the responses of microbes to these global change factors will impact marine biogeochemical cycling^3,4^.

Viruses are the most abundant microorganisms in the marine environment, with ca. 4 × 10^30^ viral particles^5,6^. Many studies have shown that viruses play an important role in marine microbial food webs^3,7–9^. The viral lysis of cells causes the release of progeny viruses and host cell components into the water, significantly increasing the cycling of dissolved organic matter (DOM). The DOM from bacterial lysates is thought to promote bacterial growth^10–12^ and affect bacterial diversity and community structures^13–15^. Recently, Jover *et al*. analysed the contents and proportions of carbon, nitrogen, and phosphorus in viral particles and proposed that the elements in viral particles cannot be ignored in marine biogeochemical cycles^16^. The decay of viral particles leads to the release of elements from the viral components to the marine DOM pool, which can be utilized by bacterioplankton and cycled within the microbial loop^17^. However, whether and how the global climate change factors (e.g., changes in temperature and light irradiation) affect natural viral population (virioplankton) is not clear^18^.

The decay processes of viruses include loss of infectivity (inactivation) and disappearance of viral particles. The rupture of the capsid shell is the major cause, leading to failure of viral adsorption onto the host cells and the ability to infect the host cells. Subject to further degradation, a decrease in the number of viral particles can be detected by flow cytometry or other techniques. In the marine environment, viral decay is dependent upon complex physicochemical and biological factors, including solar radiation, temperature, adsorption to particles, extracellular enzymes, and capsid thickness and genome size of the virus^18–21^. Solar radiation is considered one of the most important factors affecting viral inactivation and degradation^22,23^. Previous studies have shown that the ultraviolet radiation (UV) in solar radiation is the underlying factor that destroys the capsid protein and nucleic acids of viral particles^24,25^. Studies have also shown that increasing temperature enhances decay (or inactivation) rates of viral isolates in the laboratory^26^. For example, the decay rates of *Escherichia coli* viruses are positively correlated with temperature^19^. However, very limited information is available for the responses of natural viral communities to environmental (e.g., temperature or solar radiation) changes.

Therefore, for a better understanding of the role of viruses in marine microbial food webs in the ocean in the future, we investigated virioplankton decay rates and examined whether and how warming and solar radiation affects their decay. To do this, we set up three levels of experimental temperature treatments: *in situ* temperature and a 2 °C and 4 °C increase in temperature, which correspond to the present temperature and those approximately predicted for the middle and end of this century, respectively^1^. Because it is relatively well-known that UV causes both isolates and natural viral populations to decay^24,27^, in the present study, we focused on photosynthetically active radiation (PAR), which has seldom been investigated in previous studies^20^. PAR treatments at four intensities, which may reflect the possible exposure to solar irradiation of viruses in the euphotic waters^2^, were set up in the current study.

## Results and Discussion

In this study, virus and picoplankton abundances in the western Pacific Ocean were investigated with flow cytometry. Viral decay rates and their responses to changes in temperature and photosynthetically active radiation were explored with filtration techniques.

### Environmental parameters and picoplankton abundances

The western Pacific Ocean is a typical oligotrophic marine environment. The *in situ* environmental variables at the 16 stations including temperature, salinity, conductivity, density, chlorophyll *a* concentration (chl *a*), and turbidity are shown in Table S1. The chl *a* concentration was low throughout the region investigated, ranging from 0.0468 to 0.1367 mg m^−3^. The temperatures ranged from 28.10 °C to 29.80 °C, and salinity ranged from 32.07 to 34.08.

The heterotrophic bacterioplankton abundance during the investigation was 7.17 ± 0.98 × 10^5^ ml^−1^ (n = 16, ±SE), while autotrophic *Synechococcus* and *Prochlorococcus* abundances were 4.57 ± 5.85 × 10^3^ ml^−1^ (n = 16, ±SE) and 1.93 ± 1.68 × 10^4^ ml^−1^ (n = 16, ±SE), respectively. The concentration of picoeukaryotes was 4.85 ± 3.21 × 10^2^ ml^−1^ (n = 16, ±SE). The highest viral abundance was observed at the southern Station P13 (1.21 ± 0.06 × 10^7^ ml^−1^), whereas the lowest value was recorded at Station P7 (2.07 ± 0.06 × 10^6^ ml^−1^). As shown in Fig. S1, the flow cytometry analysis allowed two viral groups to be distinguished: high- and low-fluorescence viruses^28^. Low-fluorescence viruses formed the majority of the total viruses in the western Pacific Ocean, accounting for 83.93 ± 5.88% (n = 16, ±SE), on average (Table S1). The total, high-fluorescence, and low-fluorescence virus abundances were significantly positively correlated with chl *a* concentration and heterotrophic bacterial abundance (n = 16, P < 0.05, Pearson’s correlation; Table S2), suggesting that viral dynamics are closely related to and interact with both autotrophic and heterotrophic plankton^2,6^.

### Spatial variations in viral decay

**Figure 1:**
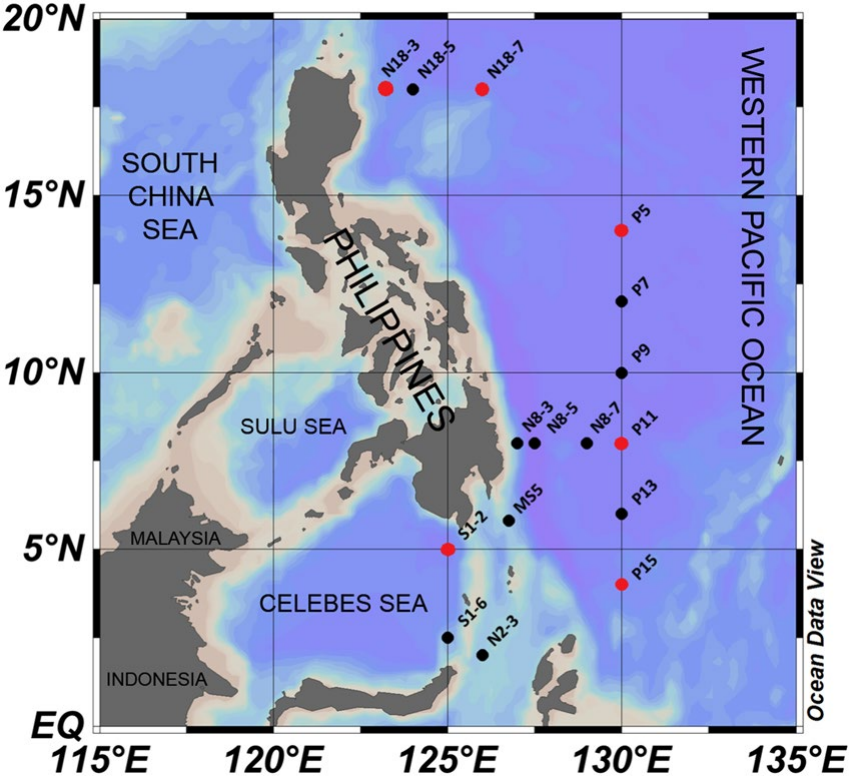
Sampling area and station locations during the National Natural Science Foundation of China cruise in the western Pacific Ocean from 25 October to 10 December 2012. The *in situ* environmental parameters and picoplankton abundances were investigated at the 16 stations (black and red dots). The viral decay experiments were explored at six stations (red dots). Cruise track was prepared using Ocean Data View software^54^ (version 4.4.1; https://odv.awi.de/).

**Figure 2:**
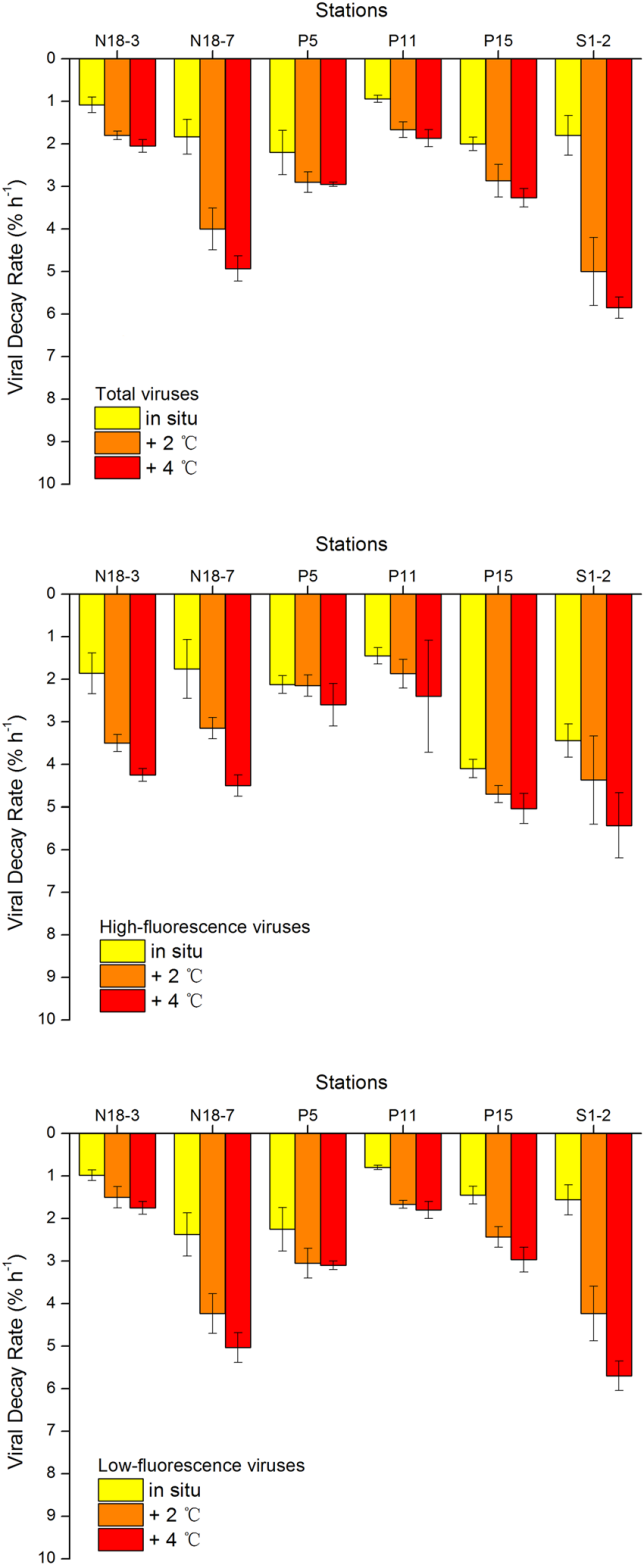
The total, high-, and low-fluorescence viruses are affected by warming (*in situ* temperature, 2 °C and 4 °C increases) in six viral decay experiment stations. Error bars indicate the standard errors calculated from triplicate sample measurements.

The decay experiment for natural virioplankton was performed at six stations in the western Pacific Ocean (Fig. 1, red dots). The viral decay rates obtained were low and were similar to those observed in other marine oligotrophic ecosystems^29–32^. Spatially, the total virus decay rates (Fig. 2) ranged from 1.08 ± 0.18% h^−1^ (corresponding to 5.92 ± 0.98 × 10^4^ ml^−1^ h^−1^; n = 3, ±SE) at Station N18-3 to 2.20 ± 0.52% h^−1^ (9.90 ± 2.34 × 10^4^ ml^−1^ h^−1^; n = 3, ±SE) at Station P5, with an average of 1.64 ± 0.21% h^−1^ (9.11 ± 1.17 × 10^4^ ml^−1^ h^−1^; n = 6, ±SE). The multivariate multiple regression analysis (DistLM-*forward*) revealed that the total virus decay rate was not significantly correlated with any of the biological or environmental factors in the investigation. Low-fluorescence virus decay rates ranged from 0.98 ± 0.13% h^−1^ (4.29 ± 0.56 × 10^4^ ml^−1^ h^−1^; n = 3, ±SE) to 2.38 ± 0.51% h^−1^ (8.23 ± 1.76 × 10^4^ ml^−1^ h^−1^; n = 3, ±SE) (average 1.57 ± 0.26% h^−1^, 6.92 ± 0.56 × 10^4^ ml^−1^ h^−1^; n = 6, ±SE). The high-fluorescence virus decay rate ranged from 1.45 ± 0.19% h^−1^ (1.73 ± 0.23 × 10^3^ ml^−1^ h^−1^; n = 3, ±SE) to 4.01 ± 0.21% h^−1^ (6.05 ± 1.05 × 10^3^ ml^−1^ h^−1^; n = 3, ±SE), with a mean value of 2.46 ± 0.43% h^−1^ (3.28 ± 0.50 × 10^3^ ml^−1^ h^−1^; n = 6, ±SE). The DistLM-*forward* analysis revealed that chl *a* explained 71.70% of the variability in high-fluorescence virus decay rate in the surface waters of the western Pacific Ocean (n = 6, P < 0.05, Table S3). This indicates a close relationship between autotrophic plankton and high-fluorescence viral dynamics because chl *a* concentration is expressed as the biomass of autotrophic plankton in general^2^. This is consistent with previous studies that autotrophic plankton (such as alga) viruses exhibit relatively higher fluorescence signals in flow cytometry analysis^33,34^. No biological or environmental factor was correlated with low-fluorescence virus decay rate, indicating that the decay behaviour of low-fluorescence viruses may be correlated with other factors, which were not measured in this investigation and need to be studied in the future. We found that the decay rates were not significantly correlated with either latitude (an indicator of solar radiation intensity) or temperature. This might be attributable to the large diel variations in solar radiation and the narrow changes in temperature in tropical and subtropical areas. Therefore, irradiance with PAR and temperature were artificially manipulated in the following experiments.

### Viral decay rates with experimental increases in temperature

**Figure 3:**
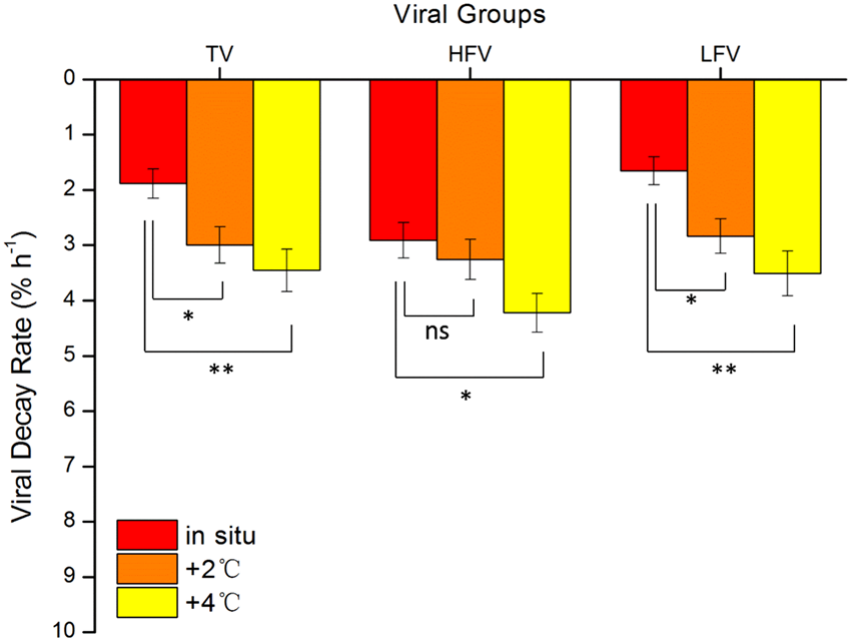
ANCOVA analysis of significant difference among average total, high-, and low-fluorescence viruses affected by warming (*in situ* temperature, 2 °C and 4 °C increases). Error bars indicate the standard errors calculated from the six experimental stations. ns, no significant difference; *P < 0.05; **P < 0.01.

The experiments of different temperature treatment impacting viral decay were performed at six stations (Fig. 1, red dots). Higher decay rates were observed for total, high-fluorescence, and low-fluorescence viruses under the 4 °C increase compared with the *in situ* temperature (Fig. 2). The maximum increase was recorded in low-fluorescence viruses at Station S1-2 (265%), and the minimum increase appeared in high-fluorescence viruses at Station P5 (22%). Significant differences in the viral decay rates were observed under + 4 °C compared to the *in situ* temperature (Fig. 3, ANCOVA, P ˂ 0.01 for total and low-fluorescence virus, P ˂ 0.05 for high-fluorescence virus). The magnitude of the increase is consistent with previous studies based on pure cultures and a meta-analysis of field observations^19,35^.

Extracellular proteases and nucleases are considered to contribute to viral decay^22,23,36^. For example, Dell’Anno *et al*. found that the viral decay rates were positively correlated with extracellular proteolytic activity, indicating proteases can destroy viral capsids, which are composed of proteins^35^. Within an appropriate range, extracellular enzymes are more active at higher temperatures, at which viral decay rates are higher. Although viruses may be more resistant to thermal stress than their hosts, many isolated viruses are very sensitive to temperature, and the inactivation events occur at a low temperature^20^. Previous studies using pure strains have also shown that the viral inactivation rates increase rapidly under warming conditions in response to thermal degradation^37,38^. However, the exact mechanism by which temperature affects virus inactivation and decay is not clear. Limited information suggested that temperature may directly affect protein stability and biomolecule elasticity of viral capsid proteins or lipid membranes, and slight alteration of temperature may affect the folding and binding of proteins and nucleic acids^20,39^.

### Viral decay rates under an experimental PAR gradient

**Figure 4:**
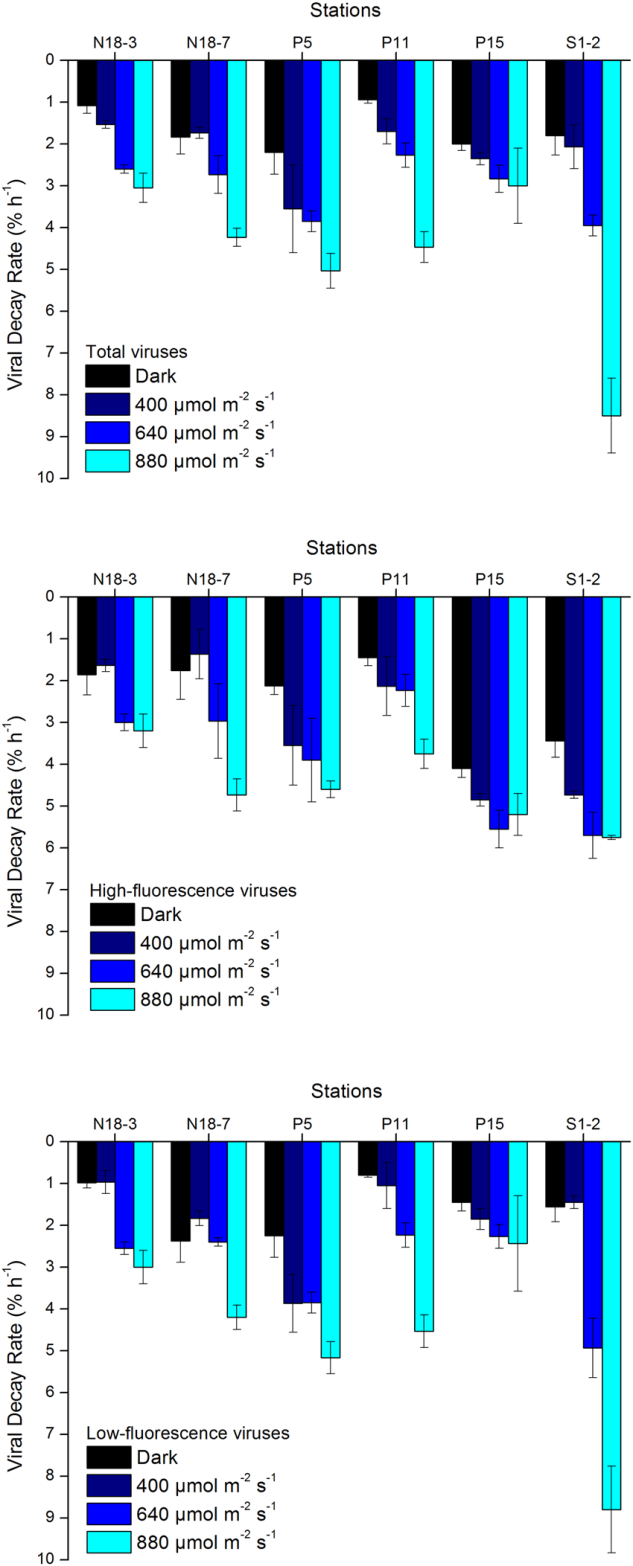
The total, high-, and low-fluorescence virus decay rates are affected by different PAR levels (dark, 400, 640, and 880 µmol m^−2^ s^–1^). Error bars indicate the standard errors calculated from triplicate sample measurements.

**Figure 5:**
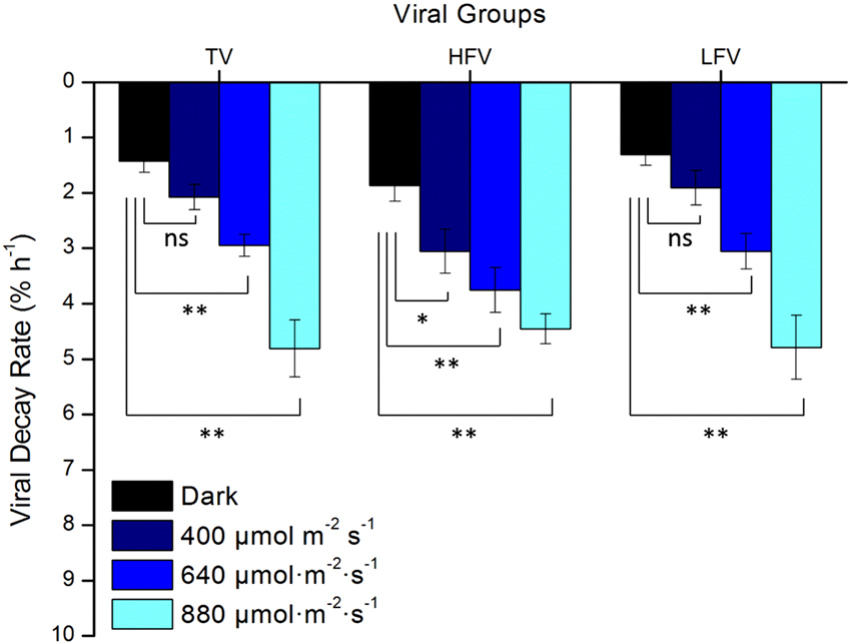
ANCOVA analysis of significant differences among average total, high-, and low-fluorescence virus decay rates affected by different PAR levels (dark, 400, 640, and 880 µmol m^−2^ s^−1^). Error bars indicate the standard errors calculated from the six experimental stations. ns, no significant difference; *P < 0.05; **P < 0.01.

In the PAR gradient experiments (Fig. 1, red dots), total, high-, and low-fluorescence viruses exhibited significantly higher decay rates under the highest PAR condition than under the lower PAR conditions and in the dark (Fig. 4), and the result of statistical analysis is shown in Fig. 5 (ANCOVA, P < 0.01 for total, high-, and low-fluorescence virus). The maximum variation appeared in low-fluorescence viruses at Station S1-2 (4.64-fold), and the minimum was observed in high-fluorescence viruses at Station P15 (0.27-fold). The UV in solar radiation is a principal factor in viral decay^24,25^. However, few studies have shown that viral decay is also affected by PAR^40,41^. Although more energy is produced by shorter wavelengths of light (such as UV), all photons carry energy, and PAR may carry enough energy to directly affect viruses by reducing their infectivity and degrading their proteins and nucleic acids, which is supported by previous studies in which blue light (434 nm) can damage DNA^42^. Traving *et al*. suggested that the light with 400–700 nm wavelengths was the major contributor to the loss of infectivity in cyanophage S-PM2^40^. Additionally, compared with the dark condition, cool white fluorescent illumination caused a significant increase in the inactivation of *Heterosigma akashiwo* virus and *Heterocapsa circularisquama* virus^41^. Beyond inactivation (i.e., infectivity loss), our study demonstrates for the first time that PAR increases marine virioplankton decay rates in natural environments.

To compare our data with previous studies (e.g., Wilhelm *et al*.^24^), we calculated the light-induced viral decay rates by subtracting the viral decay rate in the dark from that at light conditions. The light-induced (here, PAR-induced) viral decay rates were, on average, 0.53 ± 0.19% h^−1^ (n = 6, ±SE), 1.40 ± 0.20% h^−1^ (n = 6, ±SE), and 3.07 ± 0.80% h^−1^ (n = 6, ±SE) under 400, 640, and 880 μmol m^−2^ s^−1^, respectively. The solar radiation-induced (here, UV + PAR) viral decay rates integrated for the entire mixed layer above the pycnocline reported by Wilhelm *et al*.^24^ were 1.76 ± 0.45% h^−1^, higher than those obtained under low PAR in our study, demonstrating the well-known UV contribution to virioplankton decay^20^. However, the PAR-induced viral decay rates under 880 μmol m^−2^ s^−1^ observed in our study were higher than those under full light in Wilhelm *et al*.^24^, which may be explained by differences in *in situ* light conditions (e.g., our experiments were carried out at 4–18°N latitude, whereas those of Wilhelm *et al*. were at 25–27°N). Furthermore, the viral particle decay rates were always lower than the loss of infectivity measured with viral isolates in previous studies^23,24^. This is reasonable, because viruses may lose their infectivity during exposure before the destruction of the viral particles.

### Different responses of high- and low-fluorescence viruses

Higher increases in viral decay rates were always observed for low-fluorescence viruses in our experiments (Figs 3 and 5). When the +4 °C condition was compared with the *in situ* temperature, the viral decay rates increased by 75.46 ± 22.55% (n = 6, ±SE) and 120.44 ± 31.62% (n = 6, ±SE) for high- and low-fluorescence viruses, respectively. The high- and low-fluorescence virus decay rates increased 1.02 ± 0.23-fold (n = 6, ±SE) and 2.35 ± 0.76-fold (n = 6, ±SE), respectively, under the highest PAR compared with those in the dark. These suggested that high-fluorescence viruses might be more resistant to warming and PAR damage than low-fluorescence viruses. These results might be partially explained by the different density of packaged nucleic acid material (ρ_pack_) for high- and low-fluorescence viruses, where ρ_pack_ is expressed as the volume occupied by the genome divided by the internal volume of the capsid. Relatively large capsid volume of algal viruses, which usually appear as high-fluorescence populations in flow cytometry^34,43^, usually results in a relatively low ρ_pack_. For instance, the ρ_pack_ of algal viruses varies from ~0.15 to 0.4^44–49^, which are smaller than those of low-fluorescence viruses such as bacteriophages (~0.4–0.8)^19^. A higher ρ_pack_ suggests a higher internal pressure of viral particles, which may release packaged nucleic acid material when the capsid is ruptured by stressful conditions^19^. Therefore, the low-fluorescence virus with higher ρ_pack_ appears more sensitive to warming and increased PAR than high-fluorescence virus in this study.

### Biogeochemical significance of enhanced viral decay in the ocean

As an important group of microorganisms, viruses play a crucial role in marine biogeochemical cycling and global climate regulation^2^. Investigating their responses to environmental changes, such as global climate change factors, is essential to understanding viral dynamics in the marine microbial food web. The data reported herein show that warming and increased PAR can accelerate marine virioplankton decay rates. This enhanced decay will reduce the viral infection rate on their hosts, which will have significant biological, ecological, and biogeochemical consequences for marine microbial ecosystems. The increase in viral decay will also shunt more viral elements into the marine DOM pool, which contributes to the nutrient supply for surface bacterioplankton. We also observed that the acceleration of viral decay is more significant for low-fluorescence than for high-fluorescence viruses. Although the mechanism underlying this phenomenon requires further research, our study indicates that the responses of different viral groups to climate change factors differ, and, consequently, their impacts on microbial interactions in marine ecosystems differ. It is worthy to note that although the protocol for determination of viral abundance in natural samples was optimal^34,50^, the use of a preservative may affect the counting of viruses and then the decay rate. Furthermore, in a natural environment, viral decay is affected by biological factors such as protozoan grazing and virus-host interaction (e.g., viral production and cell lysis) in addition to temperature and PAR, which was artificially excluded in our study by filtration by 0.22 µm pore-size filter^2,20^. Additionally, the sudden manipulation of temperature and PAR in our experiment may not have simulated the actual conditions likely to occur in the future. The changes of temperature and other environmental factors are expected to be gradual, with fluctuation on a decadal scale. Viruses and their hosts may adapt or coevolve during this process. Therefore, further investigation with consideration of virus-host interactions and their adaption/evolution to gradually changed environmental factors is necessary for a better understanding of the response of virioplankton to global climate changes.

## Materials and Methods

### Study site and sampling

Sixteen stations were investigated during the National Natural Science Foundation of China cruise in the western Pacific Ocean (Fig. 1, black and red dots). At each station, water samples were collected from the surface (5 m depth) using a carousel water sampler carrying 12 Niskin bottles (12 litres). The samples were prefiltered through 20 µm mesh filters to remove large particles and zooplankton. A CTD profiler (SBE9/11 plus, Sea-Bird Electronics, Inc.,USA) was used to obtain salinity, temperature, conductivity, density, chl *a* concentration, and turbidity data at all stations.

### Determination of picoplankton abundance

Seawater samples (2 ml) were taken to determine picoplankton abundances. Subsamples were fixed with glutaraldehyde (0.5% final concentration), incubated at 4 °C for 15 min in the dark, flash frozen in liquid nitrogen, and then stored at −80 °C. Autotrophic picoeukaryote, *Synechococcus*, and *Prochlorococcus* abundances were determined by flow cytometry (Epics Altra II, Beckman Coulter), with scatter diagrams of side scatter vs. red fluorescence and orange fluorescence vs. red fluorescence, according to previously published methods^6,34,51^. To obtain heterotrophic bacterial abundance, the samples were stained with 1.0 × 10^−4^ SYBR Green I (v/v, final concentration, Molecular Probes), incubated for 15 min in the dark, and then analysed in a scatter diagram of red fluorescence vs. green fluorescence^6,51^. The heterotrophic bacterial abundance equalled the total microorganism abundance, subtracting the autotrophic microorganism (i.e., picoeukaryotes, *Synechococcus* and *Prochlorococcus*) abundance^51^. The samples for virus counting were diluted with Tris–EDTA buffer (pH 8.0; Sigma), stained with 5.0 × 10^−5^ SYBR Green I (v/v, final concentration), incubated at 80 °C for 10 min^34,50^, cooled to room temperature, and then analysed by flow cytometry. The yellow-green fluorescent beads with 1 µm in diameter (Molecular Probes) were added as an internal reference. All data analysis was performed with the FCS Express V3 software (De Novo Software).

### Viral decay experiment

The virioplankton decay rates were determined according to Noble and Fuhrman^10^, after the water samples had been filtered through 0.22 µm pore-size polycarbonate filters to exclude bacteria and particles >0.22 µm. The filtered water (150 ml) was then dispensed in triplicate into 50 ml aseptic tubes and incubated under each experimental condition. A temperature gradient was established with dry bath incubators (MK-20, Hangzhou Allsheng, China), *in situ* temperature, 2 °C increase, and 4 °C increase, and the light experiment was performed in a light incubator (PGX-80C, Ningbo Saifu, China) with fluorescent lamps at the *in situ* temperature. To obtain different light intensities, the tubes were either covered with aluminium foil, a sheet of white paper, or three layers of Ziploc bags or not covered. Detection by the sensor of GER1500 (Spectra Vista Corporation, USA) was covered by cutting open and flattening 50 ml tubes pasted with the different materials above, and the final light intensities in this experiment were 0 (dark), 400, 640, and 880 µmol m^−2^ s^−1^, respectively. It is important to note that the artificial light source used in the experiment, with a wavelength of 400–700 nm, provided PAR (Fig. S2).

Subsamples (1 ml) were collected every 3 h between 0 and 24 h, and the viral and bacterial abundances were determined by the method described above. Since a clear growth of bacteria at 15 h were observed in some of the incubations, which might result in infection of viruses and effect the estimation of viral decay rate, only data of 12 h incubations were applied to calculate viral decay rates in this study. The viral decay rate was calculated as the slope of the linear fitted curve of the decline in ln-transformed viral abundance during the 0–12 h experiment (see Fig. S3 for an example). The decay rate was expressed as a percent per hour by multiplying the slope by 100^22^. The decay rate calculation was applied for total, high-fluorescence, and low-fluorescence viruses.

### Statistical analysis

Pearson’s correlation analysis was used to assess the degree of correlation among the parameters investigated in SPSS Statistics 19 software (SPSS Inc., Chicago, IL, USA). Linear regression analysis was performed to assess viral decay rates at different temperatures and PAR intensities. ANCOVA analysis was performed to assess significant difference among average total, high-, and low-fluorescence viruses affected by warming (*in situ* temperature, 2 °C and 4 °C increases), and by different PAR levels (dark, 400, 640, and 880 µmol m^−2^ s^−1^). The distance-based multivariate analysis for a linear model using forward selection (DistLM-*forward*) was applied to test the relationships between viral decay rates and biotic and abiotic environmental parameters in Primer 6 software with the PERMANOVA + package (Primer-E, Plymouth, United Kingdom)^6,52,53^. The response variable was logarithmically (base 10) transformed, and the resulting data were converted into Euclidian distance similarities matrices. Fifteen variables were used to explain the variation of decay rates of high- and low-fluorescence viruses, including longitude, latitude, salinity, temperature, conductivity, density, chl *a*, turbidity, bacterial abundance, *Synechococcus* abundance, *Prochlorococcus* abundance, picoeukaryote abundance, total virus abundance, high-fluorescence virus abundance and low-fluorescence virus abundance.

## Electronic supplementary material

Supplementary information
