# Supplementary material for: Effects of temperature and photosynthetically active radiation on virioplankton decay in the western Pacific Ocean
Source: Sci Rep. 2018 Jan 24;8:1525. doi: 10.1038/s41598-018-19678-3 (PMC5784127; doi:10.1038/s41598-018-19678-3)
Supplement: Supplementary file 1 — Supplementary information [file 41598_2018_19678_MOESM1_ESM.doc]

# Effects of temperature and photosynthetically active radiation on virioplankton decay in the western Pacific Ocean

Wei Wei^1^, Rui Zhang^1^*, Lulu Peng^1^, Yantao Liang^1, 2^, Nianzhi Jiao^1^*

^1^State Key Laboratory of Marine Environmental Science, College of Ocean and Earth Sciences, Institute of Marine Microbes and Ecospheres, Xiamen University, Xiamen 361102, PR China

^2^Research Center for Marine Biology and Carbon Sequestration, Shandong Provincial Key Laboratory of Energy Genetics, Qingdao Institute of BioEnergy and BioProcess Technology, Chinese Academy of Sciences, Qingdao, 266101, PR China

*Corresponding authors: Rui Zhang (ruizhang@xmu.edu.cn); Nianzhi Jiao (jiao@xmu.edu.cn)

**
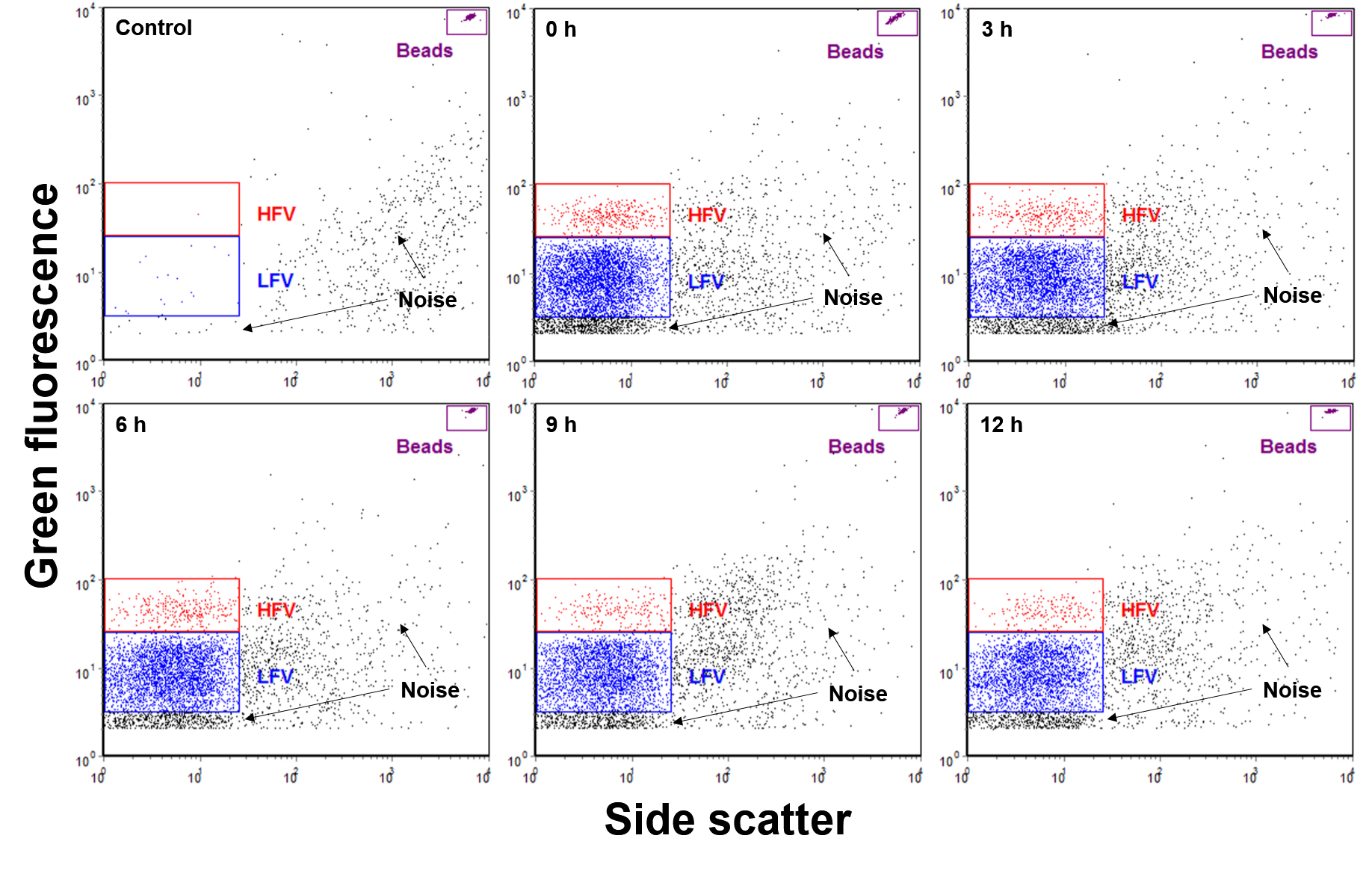
**

**Figure S1.** The scatter diagrams of Flow Cytometry analysis for high- and low-fluorescence viruses during the decay experiments (50% particles to plot). The control is 0.02 µm-filtered sample water. The beads is 1 µm in diameter with yellow-green fluorescence (Molecular Probes). HFV: High-fluorescence viruses and LFV: Low-fluorescence viruses.


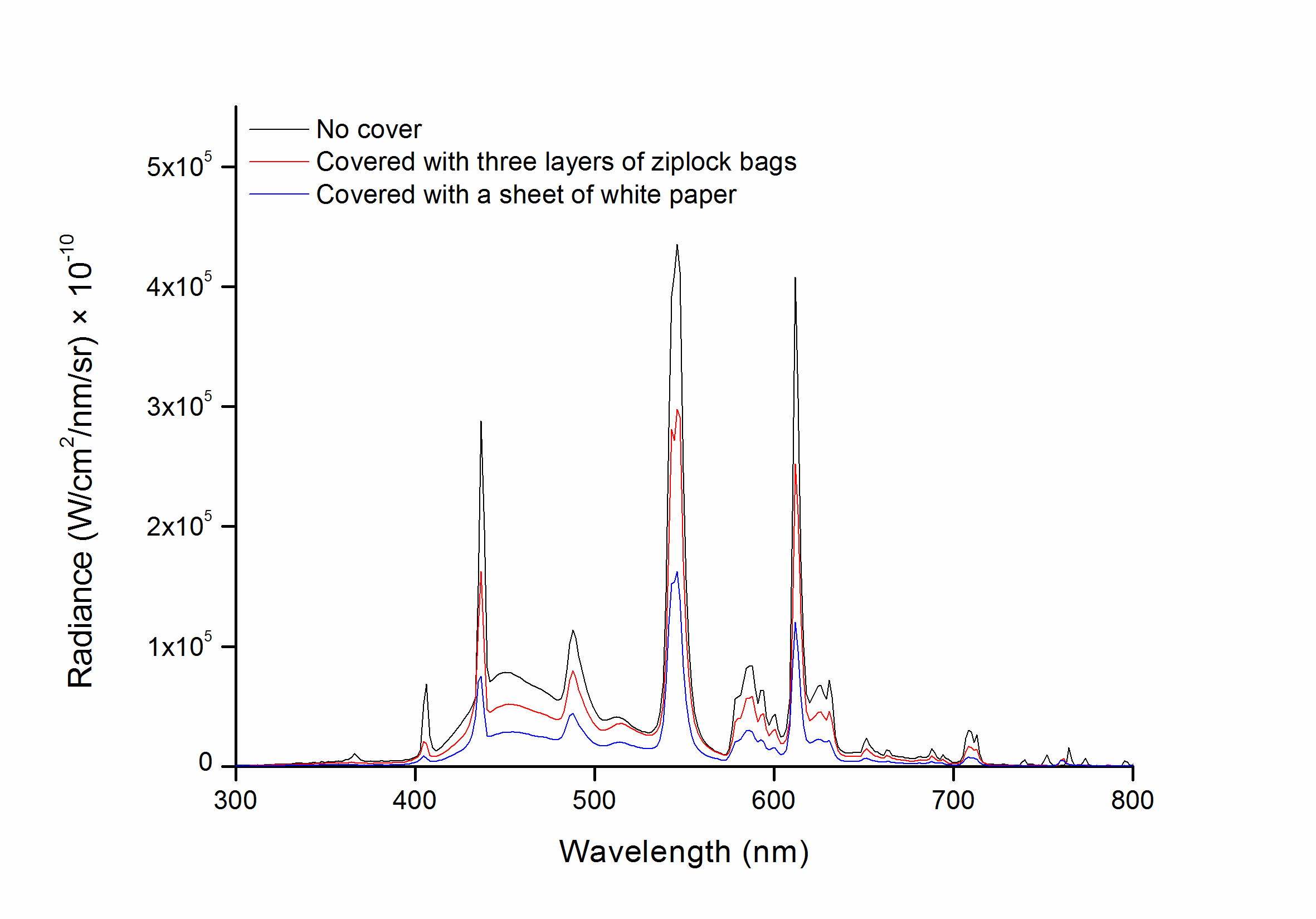


**Figure S2.** The spectrum power distribution of fluorescent lamp covered with three different materials (no cover, covered with three layers of ziplock bags and covered with a sheet of white paper) used in the experiment obtained by GER1500 (Spectra Vista Corporation, USA).


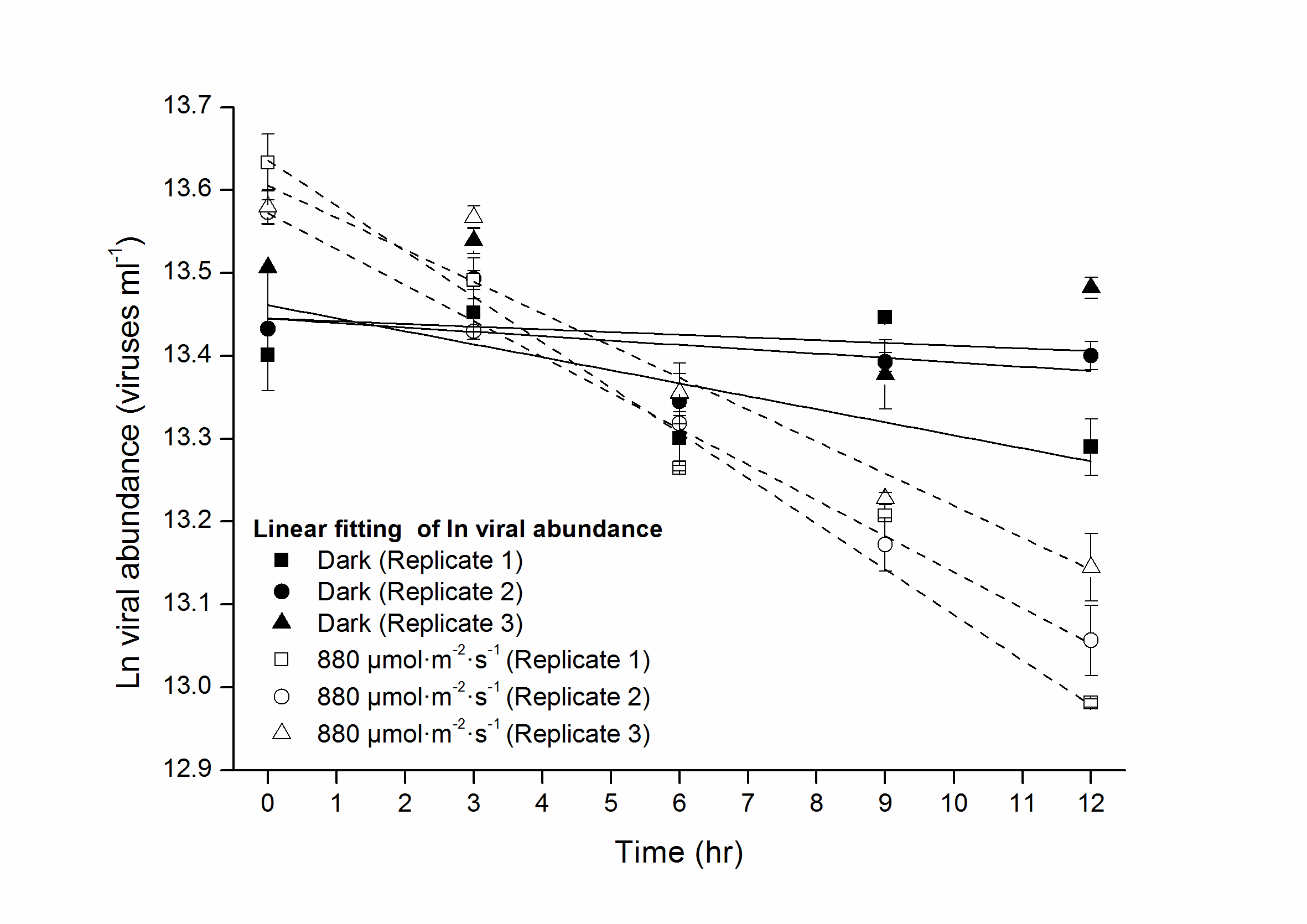


**Figure S3.** The plot of linear regression of the ln-transformed viral abundance vs. time during 0 to 12 h in viral decay experiment from triplicate incubations. The error bars are calculated by the two technical repetition of each sample.

**Table S1.** Biological and environmental parameters of the sampling stations in the western Pacific Ocean. Chl *a*: Chlorophyll *a* concentration, Bac: Bacterial abundance, Syn: *Synechococcus* abundance, Pro: *Prochlorococcus* abundance, Euk: Picoeukaryotic abundance, TV: Total virus abundance, HFV: High-fluorescence virus abundance, LFV: Low-fluorescence virus abundance, and ND: Not determined.

| Station | | Longitude | | Latitude | Salinity | Temperature | Conductivity | Density | Chl *a* | Turbidity | Bac | Syn | Pro | Euk | TV | HFV | LFV |
| --- | --- | --- | --- | --- | --- | --- | --- | --- | --- | --- | --- | --- | --- | --- | --- | --- | --- |
|  |  | (°E) | | (°N) |  | (°C) | (S/m) | (Kg/m^3^) | (10^–2^ mg/m^3^) | (10^–2^ FTU) | (10^5^ cells/ml) | (10^3^ cells/ml) | (10^4^ cells/ml) | (10^2^ cells/ml) | (10^6^ viruses/ml) | (10^6^ viruses/ml) | (10^6^ viruses/ml) |
| N18-3 | 123.25 | | 18.00 | | 32.37 | 29.00 | 5.29 | 1020.30 | 6.03 | 4.10 | 7.32 ± 0.04 | 4.91 ± 0.58 | 1.26 ± 0.07 | 8.26 ± 0.39 | 5.48 ± 0.15 | 1.05 ± 0.03 | 4.43 ± 0.014 |
| N18-5 | 124.00 | | 18.00 | | 32.86 | 28.22 | 5.34 | 1020.73 | 8.12 | 5.60 | 7.42 ± 0.11 | ND | 6.75 ± 0.10 | ND | 5.37 ±0.02 | 1.03 ± 0.12 | 4.34 ± 0.02 |
| N18-7 | 126.00 | | 18.00 | | 32.46 | 29.50 | 5.28 | 1020.40 | 4.94 | 4.70 | 6.94 ± 0.14 | 7.39 ± 0.21 | 2.14 ± 0.01 | 7.71 ± 0.25 | 4.38 ± 0.03 | 0.92 ±0.01 | 3.46 ± 0.03 |
| MS5 | 126.75 | | 5.80 | | 33.56 | 28.83 | 5.50 | 1021.07 | 4.89 | 6.20 | 6.91 ± 0.05 | 0.59 ± 0.03 | ND | 2.80 ± 0.09 | 7.30 ± 0.35 | 0.83 ± 0.02 | 6.46 ± 0.32 |
| S1-2 | 125.00 | | 5.00 | | 33.08 | 28.80 | 5.40 | 1020.80 | 5.93 | 3.80 | 6.02 ± 0.08 | 1.37 ± 0.08 | 0.49 ± 0.01 | 5.44 ± 0.11 | 4.73 ± 0.02 | 0.73 ± 0.01 | 4.00 ± 0.02 |
| S1-6 | 125.00 | | 2.50 | | 33.65 | 29.10 | 5.54 | 1021.04 | 9.55 | 13.6 | 8.46 ± 0.21 | 21.2 ± 0.40 | 2.90 ± 0.04 | 8.34 ± 0.22 | 10.4 ± 0.43 | 1.95 ± 0.07 | 8.41 ± 0.42 |
| N2-3 | 126.00 | | 2.00 | | 33.68 | 29.28 | 5.57 | 1021.00 | 8.12 | 6.70 | 7.75 ± 0.04 | 6.75 ± 0.19 | 1.29 ± 0.20 | 2.80 ± 0.05 | 7.62 ± 0.18 | 1.40 ± 0.21 | 6.21 ± 0.18 |
| P5 | 130.00 | | 14.00 | | 33.35 | 28.10 | 5.50 | 1020.80 | 5.35 | 6.00 | 6.48 ± 0.01 | 2.85 ± 0.42 | 2.09 ± 0.11 | 3.87 ± 0.09 | 4.50 ± 0.05 | 0.68 ± 0.02 | 3.82 ± 0.05 |
| P7 | 130.00 | | 12.00 | | 33.58 | 29.00 | 5.52 | 1021.02 | 5.16 | 5.60 | 6.14 ± 0.09 | 0.66 ± 0.02 | 0.48 ± 0.01 | 0.88 ± 0.01 | 2.07 ± 0.06 | 0.71 ± 0.04 | 1.37 ± 0.06 |
| P9 | 130.00 | | 10.00 | | 34.08 | 29.19 | 5.58 | 1021.34 | 4.88 | 4.20 | 6.40 ± 0.17 | 1.07 ± 0.01 | ND | 1.32 ± 0.03 | 6.48 ± 0.12 | 0.83 ± 0.02 | 5.65 ± 0.12 |
| P11 | 130.00 | | 8.00 | | 33.75 | 29.80 | 5.55 | 1021.10 | 4.68 | 4.00 | 7.56 ± 0.38 | 2.88 ± 0.04 | 2.66 ± 0.02 | 9.53 ± 0.26 | 5.34 ± 0.04 | 0.95± 0.01 | 4.38 ± 0.03 |
| P13 | 130.00 | | 6.00 | | 33.05 | 29.20 | 5.46 | 1020.56 | 13.7 | 10.1 | 8.56 ± 0.13 | 13.7 ± 0.15 | 2.45 ± 0.05 | 5.74 ± 0.15 | 12.1 ± 0.56 | 1.92 ± 0.06 | 10.1 ± 0.55 |
| P15 | 130.00 | | 4.00 | | 33.29 | 28.50 | 5.50 | 1020.70 | 9.90 | 6.60 | 8.81 ± 0.27 | 2.46 ± 0.06 | 2.76 ± 0.08 | 10.1 ± 0.31 | 8.90 ± 0.18 | 2.56 ± 0.02 | 6.34 ±0.16 |
| N8-3 | 127.00 | | 8.00 | | 32.71 | 29.05 | 5.39 | 1020.35 | 6.30 | 7.70 | 5.96 ± 0.12 | 0.59 ± 0.03 | 0.42 ± 0.01 | 1.52 ± 0.14 | 7.97 ± 0.04 | 0.66 ± 0.02 | 7.31 ± 0.04 |
| N8-5 | 127.50 | | 8.00 | | 32.07 | 28.79 | 5.44 | 1019.97 | 5.03 | 5.50 | 5.90 ± 0.02 | 0.36 ± 0.02 | 0.21 ± 0.02 | 2.26 ± 0.03 | 7.01 ± 0.15 | 0.84 ± 0.03 | 6.17 ± 0.16 |
| N8-7 | 129.00 | | 8.00 | | 33.99 | 28.92 | 5.55 | 1021.36 | 6.72 | 4.50 | 8.09 ± 0.15 | 1.70 ± 0.10 | 1.11 ± 0.14 | 2.21 ± 0.10 | 7.96 ± 0.09 | 1.02 ± 0.11 | 6.95 ± 0.08 |

**Table S2.** Pearson rank correlation coefficients and associated p-values relating biological data to environmental data obtained from surface water of western Pacific Ocean (n = 16). Chl *a*: Chlorophyll *a* concentration, Bac: Bacterial abundance, Syn: *Synechococcus* abundance, Pro: *Prochlorococcus* abundance, Euk: Picoeukaryotic abundance, TV: Total virus abundance, HFV: High-fluorescence virus abundance and LFV: Low-fluorescence virus abundance.

|  | Longitude | Latitude | Salinity | Temperature | Conductivity | Density | Chl *a* | Turbidity | Bac | Syn | Pro | Euk |
| --- | --- | --- | --- | --- | --- | --- | --- | --- | --- | --- | --- | --- |
| TV | .071 | -.610* | .095 | .095 | .252 | -.021 | .778** | .708** | .649** | .576* | .108 | .220 |
|  | .794 | .012 | .727 | .725 | .347 | .937 | .000 | .002 | .007 | .025 | .713 | .414 |
| HFV | .101 | -.461 | .120 | -.012 | .201 | .016 | .816** | .587* | .861** | .590* | .321 | .561* |
|  | .710 | .072 | .659 | .966 | .456 | .952 | .000 | .017 | .000 | .021 | .264 | .024 |
| LFV | .056 | -.592* | .080 | .113 | .242 | -.029 | .692** | .671** | .533* | .514* | .044 | .109 |
|  | .837 | .016 | .768 | .678 | .366 | .916 | .003 | .004 | .034 | .050 | .882 | .688 |
| *: correlation is significant at the 0.05 level; **: correlation is significant at the 0.01 level. | | | | | | | | | | | | |

**Table S3.** Results of the multivariate regression analysis with forward selection (DistLM-*forward*) to explain the variability in high-fluorescence virus decay rates in surface water of western Pacific Ocean. Chl *a*: Chlorophyll *a* concentration.

| Variables | Pseudo-F | P-level | Explained variance | Cumulative variance |
| --- | --- | --- | --- | --- |
| Chl *a* | 10.132 | 0.0221 | 0.71695 | 0.71695 |
